# Long-read MinION™ sequencing of 16S and 16S-ITS-23S rRNA genes provides species-level resolution of *Lactobacillaceae* in mixed communities

**DOI:** 10.3389/fmicb.2023.1290756

**Published:** 2023-12-07

**Authors:** Sandra A. Olivier, Michelle K. Bull, Mikael Lenz Strube, Robert Murphy, Tom Ross, John P. Bowman, Belinda Chapman

**Affiliations:** ^1^Tasmanian Institute of Agriculture, University of Tasmania, Hobart, TAS, Australia; ^2^Quantal Bioscience Pty Ltd., Sydney, NSW, Australia; ^3^Department of Biotechnology and Biomedicine, Technical University of Denmark, Lyngby, Denmark; ^4^Department of Biology, Section for Ecology and Evolution, University of Copenhagen, Copenhagen, Denmark

**Keywords:** *Lactobacillaceae*, amplicon sequencing, nanopore, long read sequencing, metataxonomics, microbiome, rRNA

## Abstract

The *Lactobacillaceae* are lactic acid bacteria harnessed to deliver important outcomes across numerous industries, and their unambiguous, species-level identification from mixed community environments is an important endeavor. Amplicon-based metataxonomics using short-read sequencing of partial 16S rRNA gene regions is widely used to support this, however, the high genetic similarity among *Lactobacillaceae* species restricts our ability to confidently describe these communities even at genus level. Long-read sequencing (LRS) of the whole 16S rRNA gene or the near complete rRNA operon (16S-ITS-23S) has the potential to improve this. We explored species ambiguity amongst *Lactobacillaceae* using *in-silico* tool RibDif2, which identified allele overlap when various partial and complete 16S rRNA gene and 16S-ITS-23S rRNA regions were amplified. We subsequently implemented LRS by MinION™ to compare the capacity of V3–V4, 16S and 16S-ITS-23S rRNA amplicons to accurately describe the diversity of a 20-species *Lactobacillaceae* mock community in practice. *In-silico* analysis identified more instances of allele/species overlap with V3–V4 amplicons (*n* = 43) compared to the 16S rRNA gene (*n* = 11) and partial (*n* = up to 15) or complete (*n* = 0) 16S-ITS-23S rRNA amplicons. With subsequent LRS of a DNA mock community, 80% of target species were identified using V3–V4 amplicons whilst the 16S rRNA gene and 16S-ITS-23S rRNA region amplicons resulted in 95 and 100% of target species being identified. A considerable reduction in false-positive identifications was also seen with 16S rRNA gene (*n* = 3) and 16S-ITS-23S rRNA region (*n* = 9) amplicons compared with V3–V4 amplicons (*n* = 43). Whilst the target species affected by allele overlap in V3–V4 and 16S rRNA gene sequenced mock communities were predicted by RibDif2, unpredicted species ambiguity was observed in 16S-ITS-23S rRNA sequenced communities. Considering the average nucleotide identity (ANI) between ambiguous species (~97%) and the basecall accuracy of our MinION™ sequencing protocol (96.4%), the misassignment of reads between closely related taxa is to be expected. With basecall accuracy exceeding 99% for recent MinION™ releases, the increased species-level differentiating power promised by longer amplicons like the 16S-ITS-23S rRNA region, may soon be fully realized.

## 1 Introduction

The family *Lactobacillaceae* are part of the lactic acid bacteria (LAB) group and are described as Gram-positive, non-spore-forming bacilli and coccoid species that are aerotolerant and who primarily produce lactic acid via carbohydrate metabolism (Holzapfel and Wood, 2014; Felis et al., 2015; Tian, 2019). They are ubiquitous in nature with well-known association with the gastrointestinal and urogenital tracts of animals and humans, plant material, foods, dairy, soil and water (Buron-Moles et al., 2019; Morelli and Von Wright, 2019; Liu et al., 2021). Owing to their metabolic versatility and (generally) GRAS (generally recognized as safe) status, *Lactobacillaceae* deliver important economic and social impact across numerous sectors including food, probiotic, and pharmaceutical manufacturing. They are also harnessed in biotechnological applications as ‘cell factories’ to produce a variety of compounds and to transform substrates (Buron-Moles et al., 2019; Tian, 2019; von Wright and Axelsson, 2019).

The isolation, characterization, and identification of LAB strains with novel functional potential is an increasingly important endeavor that is being actively explored from a variety of settings. However, the often complex and diverse microbial composition of environments where LAB of interest are typically found, can make the identification of species of interest challenging, especially when relying solely on culture-based methods. Culture-independent, genomic approaches can overcome these challenges and have become routine in the last decade to identify bacteria in microbial communities, especially amplicon-based metataxonomic profiling which is desired for its accessibility, cost and less onerous bioinformatics relative to metagenomics (Cao et al., 2017; Walsh et al., 2017; De Filippis et al., 2018). Short-read sequencing (SRS) of specific hypervariable regions of the 16S rRNA gene is a widely adopted amplicon-based metataxonomic approach (Starke et al., 2021; Szoboszlay et al., 2023), providing accurate and cost-efficient profiling of microbial communities. Although this approach cannot directly infer the functional capacity of a microbiome like the much more resource-intensive metagenomic approach, bioinformatic analysis (e.g., PICRUSt2) can be used to deduce predicted functionality instead (Franzosa et al., 2015; Liu et al., 2020).

Whilst LAB, chiefly including the *Lactobacillaceae*, can be arranged into distinct phylogenetic clades based on the 16S rRNA gene, because many species share more than 97% homology, the resolution between closely related species on the basis of the 16S rRNA gene can be limiting, particularly when partial 16S rRNA amplicons covering only one to two hypervariable regions are sequenced, as is the case in SRS (Vandamme et al., 2014; Wassenaar and Lukjancenko, 2014; Zheng et al., 2020; Szoboszlay et al., 2023). Metataxonomic studies of traditional fermentations utilizing SRS of the V3–V4 region of the 16S rRNA gene have noted that species-level differentiation of LAB is problematic (Deka et al., 2021; Piraine et al., 2021), with *Lacticaseibacillus casei* and *Lacticaseibacillus paracasei,* and *Secundilactobacillus collinoides* and *Secundilactobacillus paracollinoides,* specifically noted as being indistinguishable (Piraine et al., 2021). *Lactiplantibacillus plantarum* and *Lactiplantibacillus pentosus* share >99% homology and are also said to be indistinguishable based on the 16S rRNA gene (Torriani et al., 2001). The software tool, RibDif (Strube, 2021), was developed to evaluate the potential for such 16S rRNA gene overlap between species, assisting researchers to assess the feasibility of species-level metataxonomic profiling of mixed communities using partial and complete 16S rRNA gene amplicons; with RibDif it was shown that of 19 *Lactobacillus* species, *L. gasseri/paragasseri* and *L. amylovorus/ultunensis* had at least one V3–V4 16S rRNA allele overlap.

With the commercialization of long-read sequencing (LRS) technologies and an associated increase in read length beyond the ~500 bp limit of typical SRS platforms, targeted amplicon sequencing of the whole 16S rRNA gene (V1–V9), as well as the majority of the ribosomal RNA operon, *rrn* (i.e., 16S-ITS-23S), have been explored to improve bacterial, and LAB, species-level identification in complex samples (Shin et al., 2016; Benítez-Páez and Sanz, 2017; Cusco et al., 2019; Martijn et al., 2019; Brandt et al., 2020; Nygaard et al., 2020; Kinoshita et al., 2021; Seol et al., 2022). With four times the variability of the 16S rRNA coding region, sequencing the 16S-ITS-23S rRNA region can reportedly improve taxonomic resolution down to species and strain levels (Benítez-Páez and Sanz, 2017; Cusco et al., 2019). Despite being only a small part of the 16S-ITS-23S rRNA region, the internal transcribed spacer (ITS) region alone has been shown to have greater discriminatory power for LAB than 16S rRNA genes (Milani et al., 2018, 2020). Further, LAB-specific primer, ‘L5’, which amplifies part of the 16S rRNA gene (15F-687R), has been shown to be more effective in resolving LAB community composition for complex samples than the universal 16S rRNA primers (27F-1492R), and in particular, species with low relative abundance (Hou et al., 2018).

As targeted amplicon sequencing options increase with LRS, and because of the reclassification and expansion of the family *Lactobacillaceae* to comprise 33 genera (Liu and Gu, 2020; Zheng et al., 2020; Bello et al., 2022; Oliphant et al., 2022), an evaluation of various regions of the *rrn* operon to facilitate species-level identification of *Lactobacillaceae* is warranted. In this study we initially conducted *in silico* analyses using updated software tool, RibDif2 (Murphy and Strube, 2023), to identify *Lactobacillaceae* species that may be difficult to differentiate using targeted amplicon sequencing of various partial and complete 16S rRNA gene and 16S-ITS-23S rRNA regions. Further, using Oxford Nanopore Technologies’ (ONT; Oxford, United Kingdom) MinION™ LRS platform and universal primers amplifying the V3–V4, 16S rRNA gene and 16S-ITS-23S rRNA regions, mock communities comprised of 20 diverse *Lactobacillaceae* species, including candidates identified by RibDif2 that may be difficult to differentiate, were sequenced to compare the capacity of each amplicon to resolve all species of the mock community, and at the expected abundance, in practice. Together, outcomes from this study will assist in the establishment of best practice approaches for amplicon-based metaxonomic studies of LAB-rich communities.

## 2 Materials and methods

### 2.1 *In silico* evaluation of species-level differentiation of *Lactobacillaceae* based on various regions within the 16S-ITS-23S rRNA operon using RibDif2

RibDif2 (Murphy and Strube, 2023) was used to determine the feasibility to distinguish between species across the 33 genera of *Lactobacillaceae* using various partial and complete regions within the *rrn* operon. At the time of conducting this analysis (18th May 2023), the online resource, List of Prokaryotic names with Standing in Nomenclature (LPSN) (Parte et al., 2020), was used to determine the total number of validly published species across the 33 genera. In addition to the default primers, several published primers of interest targeting partial and complete 16S rRNA gene, ITS and 16S-ITS-23S rRNA regions were specified for inclusion (Table 1) using the -p/--primers argument and by providing a custom primer file. To facilitate simultaneous assessment of all amplicons of interest, including the defaults, the -w/--whole-genome argument was applied, amplifying all targets directly from the genomes. Further, the --ignore-sp argument was applied to exclude any reference sequences that were unspecified species. The default setting for clustering amplicons at 100% identity was maintained.

**Table 1:** Target amplicons and primer pair sequences used for RibDif2 *in silico* analyses.

Amplicon details	Forward		Reverse		Product size (bp)	Source
	Position	Sequence (5′–3′)	Position	Sequence (5′–3′)		
V3–V4 **16S rRNA**	341f	CCTACGGGNGGCNGCAG	805r	GACTACNNGGGTATCTAATCC	~460	RibDif2 default
V1–V9 **16S rRNA**	27f	AGRGTTYGATYMTGGCTCAG	1492r	RGYTACCTTGTTACGACTT	~1,500	RibDif2 default
V1–V9 (ONT) **16S rRNA**	27f	AGAGTTTGATCMTGGCTCAG	1492r	CGGTTACCTTGTTACGACTT	~1,500	ONT 16S Barcoding Kit (SQK-RAB204)
~V1–V4 (‘L5’) **16S rRNA**	15f	GCTCAGGAYGAACGCYGG	687r	CACCGCTACACATGRADTTC	~650	Hou et al. (2018)
**ITS rRNA** Probio-lac	‘Uni’	CGTAACAAGGTAGCCGTAGG	‘Rev’	GTYVCGTCCTTCWTCGSC	200–500	Milani et al. (2018)
**16S-ITS-23S rRNA**	27f	AGRGTTYGATYHTGGCTCAG	2241r	ACCRCCCCAGTHRAACT	~4,300	Seol et al. (2022)
23S rRNA*	Probio-lac ‘Rev’	GSCGAWGAAGGACGBRAC	2241r	ACCRCCCCAGTHRAACT	~2,000	
ITS-23S rRNA*	Probio-lac ‘Uni’	CGTAACAAGGTAGCCGTAGG	2241r	ACCRCCCCAGTHRAACT	~2,500	

^*^Primer pairs derived from primers of the same start and end points referenced for amplifying other regions (Milani et al., 2018; Seol et al., 2022). In the case of the forward primer amplifying the 23S region, this was determined by taking the reverse complement of the ITS Probio-lac ‘Rev’ primer which also marks the beginning of the 23S region.

By default, RibDif2 downloads all complete genome sequences available in the National Center for Biotechnology Information (NCBI) RefSeq database. Following initial analysis using whole genomes, analysis was repeated using all available genomes, irrespective of assembly level (i.e., contigs, scaffolds and chromosomes), using the -f/--frag argument. This increased the number of species represented in most cases and therefore provided a greater view of the potential for species overlap given nominated target regions. However, because of the low assembly level of some of the additional genomes referenced and the subsequent potential for some or all target amplicons being absent, results should be viewed as indicative rather than definitive.

RibDif2 produces an overlap summary based on each target amplicon included, identifying the number of genomes (and those amplified) and unique species included in the analysis, the number of genomes for which multiple alleles were identified, and the species that may be indistinguishable given the identification of at least one instance of target amplicon sequence (allele) overlap between them. This information can be simultaneously used to assess primer specificity.

### 2.2 Construction of mock community

A mock community comprising 20 *Lactobacillaceae* (target) species from 14 genera (Table 2) was assembled by two separate approaches based on an approximately equal contribution of (1) cells from each species to mimic a microbiome sample for which DNA copies are not accounted for, and (2) DNA copies from each species for a more controlled sample. Species were selected with a view to include as many as possible that had been identified by RibDif2 to overlap with closely related species based on the V3–V4 and/or V1–V9 16S rRNA region (*n =* 10). The balance of the mock community was selected to provide diversity of other *Lactobacillaceae* genera.

**Table 2:** *Lactobacillaceae* species in the mock community and DNA-MC assembly details.

Species	Source and/or reference number^*^	Other reference numbers^*^	NCBI RefSeq assembly number for actual or representative strain^^^	Genome size (Mb)	GC content (%)	16S rRNA copies^#^	Cell suspension DNA conc. (ng/μL)	Volume in DNA-MC (μL)
*Fructilactobacillus fructivorans*	Food sample	–	GCF_009496955.1 ^O^	1.4	39.0	4^M^	24.50	0.62
*Lacticaseibacillus casei*	UNSW culture collection	–	GCF_000829055.1^R^	3.0	47.5	5^M^	20.25	1.60
*Lacticaseibacillus paracasei*	Commercial culture	CSL IMC502	GCF_000155515.2 ^R^	3.0	46.0	5^M^	7.65	4.23
*Lactiplantibacillus paraplantarum*	NRRL B-23115	DSM 10667	GCF_001435655.1 ^A^	3.4	43.5	5^M^	13.40	2.74
*Lactiplantibacillus pentosus*	NRRL B-227	DSM 20314	GCF_003641185.1 ^A^	3.7	46.0	5^A^	6.89	5.80
*Lactiplantibacillus plantarum*	Commercial culture	CSL LB931	GCF_009913655.1 ^R^	3.2	44.5	5^M^	12.40	2.79
*Lactobacillus amylovorus*	NRRL B-4540	DSM 20531	GCF_002706375.1 ^A^	2.2	37.5	5^A^	6.04	3.93
*Latilactobacillus sakei*	Commercial culture	CH B-LC-007	GCF_009676365.1 ^R^	2.0	41.0	7^M^	15.45	1.40
*Lentilactobacillus buchneri*	Unilever Research Lab	VBLLa 18.01	GCF_018314255.1 ^R^	2.5	44.0	5^M^	9.83	2.75
*Leuconostoc citreum*	NRRL B-1501	ATCC 10882	GCF_007954785.1^R^	1.9	38.5	4^M^	3.78	5.43
*Leuconostoc lactis*	Commercial culture	CH B-LC-007	GCF_007954605.1 ^R^	1.8	43.0	4^M^	4.77	4.07
*Leuconostoc mesenteroides* subsp. *mesenteroides*	NRRL B-1118	ATCC 8293	GCF_000014445.1 ^A^	2.1	37.5	4^A^	4.63	4.90
*Levilactobacillus brevis*	Commercial culture	CSL SP48	GCF_003813165.1 ^R^	2.5	45.5	5^M^	7.03	3.84
*Ligilactobacillus salivarius*	Food plant sample	-	GCF_900094615.1 ^R^	2.0	32.5	7^M^	15.15	1.42
*Limosilactobacillus fermentum*	Commercial culture	CSL CS57	GCF_022819245.1 ^R^	2.0	51.5	5^M^	12.55	1.72
*Limosilactobacillus reuteri*	Commercial culture	CSL LR92	GCF_000016825.1^T^	2.0	38.5	6^M^	8.85	2.68
*Liquorilactobacillus mali*	NRRL B-4563	DSM 20444	GCF_001436535.1 ^A^	2.6	36.0	6^M^	9.21	3.05
*Loigolactobacillus coryniformis*	Food sample	–	GCF_002706425.1 ^R^	3.0	43.0	5^M^	12.90	2.51
*Pediococcus acidilactici*	Food sample	–	GCF_013127755.1 ^R^	2.0	42.0	5^M^	6.19	3.49
*Weissella confusa*	NRRL B-1064	DSM 20196	GCF_001436895.1 ^A^	2.2	44.5	9^M^	4.02	5.91

^*^UNSW-University of New South Wales, Australia; NRRL-Agriculture Research Service culture collection; CSL-Centro Sperimentale del Latte; DSM-German Collection of Microorganisms and Cell Cultures GmbH; CH-Christian Hansen; ATCC-American Type Culture Collection. ^^^Species with a complete genome submitted in the NCBI RefSeq database are marked with an ^A^ and all information regarding genome size and GC content are based on those sequences. For all other species, the complete reference ^R^, Type strain ^T^, or only available ^O^ genome was consulted to derive indicative values. ^#^As reported in the *rrn*DB (Stoddard et al., 2015) for actual RefSeq genome ^A^ or an indicative, median value as reported for all referenced genomes ^M^.

Species were recovered from −80°C glycerol stocks in Lactobacilli MRS broth (BD Difco, United States) at 30 or 37°C (*Lactobacillus amylovorus, Ligilactobacillus salivarius, Limosilactobacillus fermentum* only) for 48 ± 2 h, under anaerobic conditions (Anaerogen, Oxoid, United Kingdom). Cultures were diluted or concentrated (4,000 x *g*) in phosphate buffered saline to achieve ~10^8^ cfu/mL as assessed by microscopy (Helber counting chamber), and equal volumes of each were combined to prepare a cell cocktail. DNA was extracted from 1.8 mL of the cell cocktail, and from individual culture suspensions using the Qiagen DNeasy PowerFood Microbial Kit (Qiagen, Germany) and according to its Quick-Start Protocol, except for the bead beating step which implemented a Mini-Beadbeater-24 (Biospec, United States) for 1 min. Further, DNA was eluted in 50 μL Solution EB. The concentration of DNA for each extraction was quantified using a Qubit fluorometer (Invitrogen, United States). DNA from the cell cocktail constituted the cell mock community (CELL-MC), and the DNA mock community (DNA-MC) was assembled by adding the volume of each species’ extracted DNA that would contribute ~10^6^ copies of DNA given the actual or estimated size of the genome (Table 2).

### 2.3 PCR amplification of V3-V4, 16S and 16S-ITS-23S rRNA regions

Nanopore barcoded V3–V4 and 16S-ITS-23S rRNA amplicons were amplified by PCR in two stages with reference to previously described methodology (Kerkhof et al., 2017; Cusco et al., 2019). CELL-MC and DNA-MC DNA was first amplified using ONT-tagged forward (ONT tag 5’-TTTCTGTTGGTGCTGATATTGC-) and reverse (ONT tag 5’ACTTGCCTGTCGCTCTATCTTC-) primers as per Table 1. Template DNA (5 ng), 12.5 μL 2X LongAmp HotStart Taq (New England Biolabs, United States), 1 μL of each primer (10 mM) and nuclease free water up to a final reaction mix volume of 25 μL was prepared. Initial denaturation occurred at 94°C for 30 s, followed by 30 cycles of 94°C/20 s, 55°C/30 s and 65°C for 30 s (V3–V4) or 3.5 min (16S-ITS-23S). Final annealing occurred at 65°C for 5 min (V3–V4) or 10 min (16S-ITS-23S). PCR products were confirmed by gel electrophoresis and the DNA quantified using a Qubit fluorometer. Amplified products were then barcoded using the ONT Barcoding Kit (SQK-PBK004). Template DNA (200 ng), 12.5 μL 2X LongAmp HotStart Taq, 0.75 μL of unique barcode and nuclease free water up to a final reaction mix volume of 25 μL was prepared. The PCR cycle was as previous, except 15 cycle repeats were used. Barcoded amplicons (25 μL) were cleaned up using 30 μL MagBio HighPrep PCR magnetic beads (MagBio Genomics, United States) according to the manufacturer’s instructions for use in tubes, except DNA was eluted in 10 μL warmed (50°C) 10 mM Tris–HCl pH 8.0 with 50 mM NaCl.

The complete 16S rRNA gene was amplified from the CELL-MC and DNA-MC using the ONT 16S Barcoding Kit 1–12 (SQK-RAB204). Template DNA (5 ng), 12.5 μL 2X LongAmp HotStart Taq, 0.5 μL of unique barcode and nuclease free water up to a final reaction mix volume of 25 μL was prepared. Initial denaturation occurred at 95°C for 1 min, followed by 25 cycles of 95°C/20 s, 55°C/30 s and 65°C/2 min. Final annealing occurred at 65°C for 5 min. Barcoded amplicons were cleaned up as previously described.

### 2.4 Nanopore sequencing of mock communities

Sequencing was undertaken separately for each target amplicon due to differences in sequence length. In each case, “half-reactions” were adopted; barcoded CELL-MC and DNA-MC DNA were pooled in equal proportions, targeting a combined concentration of 50 fmol in 5 μL. To prepare the library for sequencing, pooled DNA was combined with 0.5 μL RAP (from respective barcoding kits), 17 μL sequencing buffer and 12.50 μL loading beads (ONT Flow Cell Priming kit; EXP-FLP002), and 2.25 μL nuclease free water. The library was loaded and sequenced on an ONT flow cell (FLO-MIN106, R9.4.1), fitted to the MinION™ Mk1C (MC-113090), as per the manufacturer’s instructions. MinKNOW software (version 22.12.5) was used for raw data collection. Sequencing ran until each barcoded sample reached at least ~40,000 reads.

### 2.5 Data analysis workflow

The Fast5 files generated for each sample were basecalled, demultiplexed, trimmed (barcodes and adapters) and filtered to a minimum quality score of 10 using Guppy (version 6.5.7) in super accuracy mode (Guppy-SUP), retaining reads with barcodes on one end. ToulligQC (version 2.2.1) was used to assess the quality of reads and the number retained post-Guppy. Nanofilt (version 2.8.0) filtered out reads that did not fall within the desired length range for each amplicon: 400–500 bp for V3–V4, 1,000–2,000 bp for 16S, and 3,800–4,500 bp for 16S-ITS-23S, and seqtk (version 1.4 - r122) was implemented to randomly select 39,205 reads across amplified samples to match the V3–V4 DNA-MC sample, except for the 16S-ITS-23S CELL-MC sample which had 21,931 reads post-filtering. Nanoplot (version 1.41.3) was used to determine the mean quality and length of reads that would be used for downstream analysis.

For optimized taxonomic assignment of ONT generated reads, the Emu community profiling tool (version 3.4.4; Curry et al., 2022) was implemented which generated relative abundance and read assignment distribution (−-keep-counts and --keep-read-assignments argument) tables. The default Emu database was used for V3–V4 and 16S rRNA read classification. For classification of 16S-ITS-23S rRNA reads, a custom database was generated with Emu’s build-database function using sequences from the MIrROR 16S-ITS-23S rRNA reference database which represents 9,485 species derived from 43,653 genomes and 97,781 rRNA operon sequences (Seol et al., 2022).

For each amplicon, the relative abundance (RA, %) of identified taxa in both CELL-MC and DNA-MC samples was derived from the Emu output; false positive species detected at <1% RA were considered collectively as one group rather than reporting them individually. The relative difference (RD) in RA for each mock community target species, compared to the theoretical RA (TRA; i.e., 5%), was determined as follows (Park et al., 2021):


$$RD=\frac{RA-TRA}{TRA}$$

Positive and negative RD values indicated over- and underrepresentation of species, respectively, where RD values ranging −0.5 to −0.9 or 0.5 to 0.9 represented a moderate difference whilst ≤ − 1.0 or ≥ 1.0 represented a considerable difference. Alpha diversity was assessed at the species level for all samples according to absolute richness (total species, *S*), as well as by Shannon diversity (*H*) and Pielou’s evenness [*J* = *H*/ln(*S*)] indices; given the known, theoretical composition of both mock communities, the maximum possible value for each alpha diversity metric was calculated as *S* = 20, *H* = 3.00 and *J* = 1.00.

## 3 Results

### 3.1 *Lactobacillaceae* species with overlapping alleles according to partial and complete 16S and 16S-ITS-23S rRNA amplicons

Of the 33 genera (representing 382 species) classified in the family *Lactobacillaceae* at the time of analysis (Parte et al., 2020), 27 (representing 376 species) were eligible and relevant for RibDif2 based on having at least two member species; *Acetilactobacillus*, *Convivina*, *Dellaglioa*, *Holzapfeliella*, *Nicoliella*, and *Paralactobacillus* were therefore excluded from all analyses. Analysis referencing whole genome sequences (WGS; *n* = 953) in RefSeq further reduced the number of eligible genera to 20 (representing 131 species); *Agrilactobacillus*, *Amylolactobacillus*, *Fructobacillus*, *Furfurilactobacillus*, *Lapidilactobacillus*, *Periweissella*, and *Schleiferilactobacillus* were excluded due to only one genome or species having WGS available. Repeat analysis referencing genomes of any assembly level (AGS; *n* = 6,761) in RefSeq reinstated these genera and considerably increased the number of species represented overall (*n* = 204–327, depending on amplicon), providing an increased opportunity to discover species that are potentially indistinguishable when amplifying specific regions in the rRNA region for metataxonomic purposes. A summary of key metrics output from RibDif2 analysis is provided in Supplementary Tables S1, S2, based on WGS and AGS, respectively.

Overlap of the sequence of even one target amplicon (allele) shared between species from the same genus indicates the potential for these species to be indistinguishable if present in mixed community samples undergoing amplicon-based metataxonomic investigation; this may result in the misclassification of one arising from misassignment of reads to the other, or vice versa. RibDif2 analysis of *Lactobacillaceae* genera with respect to partial and complete 16S and 16S-ITS-23S rRNA regions (Table 3) shows considerably greater potential for misclassification based on V3–V4 amplicons (~460 bp), with up to 43 overlapping species from 10 genera identified. Using sequences of the whole 16S rRNA gene (~1,500 bp) reduces species ambiguity with up to 11 overlapping species from 5 genera identified, and the mid-length sized ‘L5’ amplicon (Hou et al., 2018), which roughly spans the V1–V4 rRNA region (~650 bp), offering comparable outcomes.

**Table 3:** *Lactobacillaceae* species with overlapping (OL) sequences of given target amplicons as determined with RibDif2, drawing on whole (WGS) and all assembly level (AGS) genomes from NCBI.

Genus	Species	V3–V4		V1–V9*		L5		ITS		23S		ITS-23S		16S-ITS-23S	
		WGS	AGS	WGS	AGS	WGS	AGS	WGS	AGS	WGS	AGS	WGS	AGS	WGS	AGS
*Apilactobacillus*	*apinorum*								OL						
	*kunkeei*														
*Companilactobacillus*	*pabuli*	OL	OL												
	*farciminis*														
	*crustorum*														
	*zhachilii*														
	*heilongjiangensis*														
	*paralimentarius*		OL												
	*kimchii*														
	*bobalius*														
*Fructilactobacillus*	*cliffordii*	OL	OL												
	*myrtifloralis*														
	*carniphilus*														
*Lacticaseibacillus*	*paracasei*	OL	OL		OL		OL				OL				
	*casei*							OL	OL						
	*zeae*														
	*chiayiensis*														
*Lactiplantibacillus*	*paraplantarum*	OL	OL					OL	OL						
	*argentoratensis*									OL	OL	OL	OL		
	*plantarum*			OL	OL	OL	OL								
	*pentosus*														
	*fabifermentans*														
*Lactobacillus*	*amylovorus*	OL	OL												
	*ultunensis*														
	*gasseri*	OL	OL	OL	OL	OL	OL	OL	OL		OL				
	*paragasseri*														
	*jensenii*		OL				OL								
	*mulieris*														
*Leuconostoc*	*falkenbergense*	OL	OL												
	*pseudomesenteroides*														
	*mesenteroides*	OL	OL												
	*suionicum*														
	*garlicum*	OL	OL	OL	OL	OL	OL		OL						
	*lactis*														
	*citreum*														
*Ligilactobacillus*	*animalis*	OL	OL												
	*murinus*														
*Limosilactobacillus*	*portuensis*	OL	OL												
	*reuteri*														
*Liquorilactobacillus*	*hordei*		OL												
	*mali*														
*Weissella*	*cibaria*	OL	OL												
	*confusa*														
	*ceti*	OL	OL		OL		OL		OL		OL				
	*tructae*														
Total species amplified		131	372	131	356	133	361	131	228	131	352	131	232	131	204
Total species with overlapping amplicons		34	43	6	11	6	13	8	15	2	8	2	2	0	0

^*^Represents the combined, identical results determined using the default primers for V1–V9 in RibDif2 and the ONT 16S primers.

Including the sequence of the ITS to 23S region of the *rrn* operon with the 16S rRNA gene further resolves the discrimination of *Lactobacillaceae* species (Table 3). Except for the ITS rRNA region (~200–500 bp) in isolation, which introduces species overlap not otherwise observed (*Apilactobacillus apinorum/kunkeei* and *Lacticaseibacillus casei*/*zeae*) and up to 15 species’ overlaps overall, 23S (~2,000 bp) and ITS-23S (~2,500 bp) rRNA amplicons reduce the observation of species overlap to up to 8 and 2 instances, respectively. However, amplicons spanning the 16S-ITS-23S rRNA region (~4,500 bp) had no overlapping sequences and suggests all previously ambiguous/overlapping *Lactobacillaceae* species can be distinguished.

This comprehensive analysis of potential *Lactobacillaceae* species’ overlap with respect to amplicons that are established (V3–V4) or emerging (16S and 16S-ITS-23S) sequence targets for metataxonomic studies, highlights the key genera and species for which there may be ongoing challenges for species-level identification, even with longer amplicons. In the inevitable transition to full 16S rRNA amplicon sequencing, in lieu of partial 16S rRNA regions, *Lactobacillus gasseri/paragasseri, Lacticaseibacillus casei/paracasei, Lactiplantibacillus plantarum/pentosus*, *Leuconostoc garlicum/lactis/citreum*, and *Weissella ceti/tructae* may continue to be problematic to definitively identify. Only with sequencing of the majority of the *rrn* operon via the 16S-ITS-23S rRNA region do the results here suggest that these species can be distinguished.

The inclusion of genomes from all assembly levels notably increased the number of species included in each analysis which subsequently led to the identification of overlapping species not otherwise observed. However, consideration must be given to the possibility that some incomplete genomes deposited in NCBI may not yet be accurately identified if identification was based on short rRNA regions. The results here point to an increased chance of misidentification for some species when based on shorter rRNA amplicons.

Based on RibDif2 outcomes, the V3–V4, 16S rRNA and 16S-ITS-23S rRNA regions were selected as relevant amplicons on which to compare community profiles for *Lactobacillaceae* mock communities. Further, where available, species that had been identified to overlap with respect to the V3–V4 and/or 16S rRNA region were selected for inclusion in the mock community to determine if these could be resolved to species level by MinION™ LRS when amplified with 16S rRNA and/or 16S-ITS-23S rRNA primers, respectively.

### 3.2 Primer specificity and database representation for mock community target species

The RibDif2 output provided insight into the specificity of each primer pair with respect to *Lactobacillaceae* species. As part of method development, this data was used to confirm that all species in the mock community could be amplified using the V3–V4, 16S and 16S-ITS-23S rRNA primers, with provision for one mismatch and one insertion.

Further, by searching the Emu 16S and MirROR database FASTA and taxonomy files, it was confirmed that all species in the mock community were represented with at least one amplicon sequence (Supplementary Table S3). Supplementary Table S3 also indicates which strains were specifically represented in each database as identified by searching for the NCBI RefSeq assembly or culture collection reference number (as noted in Table 2).

### 3.3 Basecall accuracy and read count metrics

Although sequencing ran until each barcoded sample reached ~40,000 reads, filtering by Nanofilt for read length notably reduced the number of reads for taxonomic classification for V3–V4 (~28% loss) and 16S-ITS-23S (~44% loss) rRNA amplicons (Table 4), whereas ~98% of 16S rRNA amplicons were retained; this read loss was unique to samples prepared by the 2-step PCR protocol (i.e., V3–V4 and 16S-ITS-23S) and was attributed to the majority of these discarded reads being too short compared to the desired target read length range. This read loss would have no impact on the reliability of downstream taxonomic assignment. The average (± standard deviation) basecall accuracy for normalized reads was 96.49 ± 0.11% (Table 4).

**Table 4:** Read metrics for CELL-MC and DNA-MC amplicons.

Metric	CELL-MC			DNA-MC		
	V3–V4	16S	16S-ITS-23S	V3–V4	16S	16S-ITS-23S
Reads post-Guppy	93,963	134,887	38,790	52,790	68,273	67,277
Reads post-Nanofilt	70,250	132,171	21,931	39,205	67,506	39,858
Reads post-normalization	39,205	39,205	21,931	39,205	39,205	39,205
Mean read length	463	1,472	4,087	463	1,484	4,084
Mean PHRED score	14.70	14.50	14.40	14.70	14.60	14.40
Estimated read accuracy (%)	96.61	96.45	96.37	96.61	96.53	96.37

### 3.4 Taxonomic classification of *Lactobacillaceae* mock communities by targeted V3–V4, 16S and 16S-ITS-23S rRNA amplicon sequencing with MinION™

The ability to resolve the species-level identity of all *Lactobacillaceae* in a mock community comprised of 20 diverse and closely related species was assessed and compared using MinION™ long-read amplicon sequencing targeting the V3–V4, 16S and 16S-ITS-23S rRNA regions (Table 5; Supplementary Table S4 presents raw abundance data). An additional variable of mock community preparation was introduced with either an equal contribution of (1) cells (~10^8^ cfu each) or (2) DNA copies (~10^6^ each) to represent (1) a mixed community sample for which estimated DNA copy per species cannot be corrected for during sample preparation, and (2) a sample for which potential PCR amplification bias attributed to DNA copy disparity between species could be minimized to better isolate differences in community profile outcomes attributed to target amplicon.

**Table 5:** Metataxonomic analysis of Cell and DNA mock communities according to target amplicon, reported as relative abundance (RA), and relative difference (RD)* compared to theoretical abundance (5%)^, for each species.

Species in mock community	CELL-MC						DNA-MC					
	V3–V4		16S		16S-ITS-23S		V3–V4		16S		16S-ITS-23S	
	RA (%)	RD	RA (%)	RD	RA (%)	RD	RA (%)	RD	RA (%)	RD	RA (%)	RD
*Fructilactobacillus fructivorans*	15.54	2.1	18.83	2.8	22.16	3.4	4.44	−0.1	6.27	0.3	6.44	0.3
*Lacticaseibacillus casei*	0.73	−0.9	8.18	0.6	0.25	−0.9	1.35	−0.7	11.72	1.3	0.29	−0.9
*Lacticaseibacillus paracasei*	16.72	2.3	10.71	1.1	17.72	2.5	18.89	2.8	8.60	0.7	19.81	3.0
*Lactiplantibacillus paraplantarum*	ND^#^	ND	2.58	−0.5	2.63	−0.5	ND	ND	4.63	−0.1	4.30	−0.1
*Lactiplantibacillus pentosus*	ND	ND	0.04	−1.0	0.05	−1.0	ND	ND	ND	ND	0.12	−1.0
*Lactiplantibacillus plantarum*	9.65	0.9	5.81	0.2	5.61	0.1	12.87	1.6	7.00	0.4	8.11	0.6
*Lactobacillus amylovorus*	1.05	−0.8	1.35	−0.7	0.68	−0.9	1.79	−0.6	2.43	−0.5	1.18	−0.8
*Latilactobacillus sakei*	11.03	1.2	12.62	1.5	13.28	1.7	8.62	0.7	9.18	0.8	9.85	1.0
*Lentilactobacillus buchneri*	2.32	−0.5	3.02	−0.4	3.13	−0.4	3.65	−0.3	5.34	0.1	4.87	0.0
*Leuconostoc citreum*	ND	ND	1.37	−0.7	1.27	−0.7	0.05	−1.0	1.84	−0.6	1.47	−0.7
*Leuconostoc lactis*	0.05	−1.0	2.12	−0.6	2.24	−0.6	0.06	−1.0	3.04	−0.4	2.64	−0.5
*Leuconostoc mesenteroides*	0.04	−1.0	1.81	−0.6	1.26	−0.7	0.07	−1.0	2.21	−0.6	1.63	−0.7
*Levilactobacillus brevis*	2.46	−0.5	2.25	−0.5	1.55	−0.7	4.02	−0.2	3.74	−0.3	3.15	−0.4
*Ligilactobacillus salivarius*	11.21	1.2	7.62	0.5	5.17	0.0	6.83	0.4	5.24	0.0	3.34	−0.3
*Limosilactobacillus fermentum*	4.67	−0.1	3.90	−0.2	1.61	−0.7	4.32	−0.1	3.37	−0.3	1.34	−0.7
*Limosilactobacillus reuteri*	5.69	0.1	4.74	−0.1	5.75	0.2	6.96	0.4	5.36	0.1	5.83	0.2
*Liquorilactobacillus mali*	1.35	−0.7	4.47	−0.1	4.10	−0.2	1.02	−0.8	7.23	0.4	6.94	0.4
*Loigolactobacillus coryniformis*	3.57	−0.3	4.40	−0.1	3.94	−0.2	4.03	−0.2	5.23	0.0	4.65	−0.1
*Pediococcus acidilactici*	2.55	−0.5	0.85	−0.8	1.40	−0.7	4.22	−0.2	1.55	−0.7	2.64	−0.5
*Weissella confusa*	ND	ND	1.65	−0.7	0.75	−0.9	0.08	−1.0	5.06	0.0	2.14	−0.6
*False-positive species identified at > 1% with at least one amplicon*												
*Lacticaseibacillus rhamnosus*	0.93		0.75		0.25		1.15		ND		0.32	
*Lacticaseibacillus zeae*	ND		ND		4.10		ND		ND		7.58	
*Latilactobacillus fuchuensis*	2.24		ND		ND		1.25		ND		ND	
*Liquorilactobacillus hordei*	2.16		0.05		ND		4.53		ND		0.05	
*Weissella cibaria*	1.40		0.03		ND		4.15		ND		0.07	
*Additional false-positive species identified at < 1% abundance*												
Number	33		6		4		39		3		5	
Combined RA (%)	4.66		0.87		1.11		5.66		0.97		1.25	

^*^The theoretical proportion of each species in the mock community was determined based on an estimated equal contribution of cells or DNA copies in the mock community. ^Positive and negative RD indicate over and under representation of species, respectively. RD values −0.5 to −0.9 (pale blue) and 0.5 to 0.9 (pale pink) represent a moderate difference, whilst ≤ − 1.0 (dark blue) or ≥ 1.0 (dark pink) represent a considerable difference. ^#^Species not detected (ND); undetected mock community species are highlighted darkest blue.

As anticipated by the outcomes of the RibDif2 analysis, sequencing of the V3–V4 rRNA region resulted in the greatest incidence of unidentified target species in the CELL-MC (20%) and DNA-MC (10%) samples (Table 5). With regards to the determined vs. theoretical RA of target species (Table 5), for the CELL-MC, 60% were underrepresented (RD ≤ -0.5) and 25% were overrepresented (RD ≥0.5). This was marginally improved using the DNA copy-corrected DNA-MC with 45 and 15% of the population under- and over-represented, respectively, which was also reflected in an improvement in the Shannon diversity index (*H*) from 2.71 to 2.85 (Table 6). However, calculated evenness (Table 6) did not appreciably change (*J* = 0.68 to 0.70) which was symptomatic of the disproportionate number of underrepresented and, largely, falsely identified species in both mock communities (the complete list of species is available in Supplementary Table S4). The false identification of 37 and 43 additional species from the *Lactobacillaceae* family in the CELL-MC (11.4% RA) and DNA-MC (16.7% RA), respectively, resulted in far greater richness (*S =* 53 and 59 species, respectively) than expected (i.e., *S =* 20), however, a long tail of low-abundant species is an outcome anticipated by the authors of the Emu classification tool (Curry et al., 2022) which can be managed by increasing the default abundance threshold (0.01%). Applying an abundance threshold of 0.1% reduced false positive species to 19 in both mock communities, increasing evenness to 0.70 and 0.79 for CELL-MC and DNA-MC, respectively, however, previously detected low-abundant target species were no longer identified, reducing the proportion of target species identified to 70 and 75%, respectively (complete data not shown). Alternative bioinformatic tools that may have improved V3–V4 rRNA amplicon analysis were not sought.

**Table 6:** Alpha diversity metrics for Cell and DNA mock communities according to target amplicon.

Metric	Theoretical value	CELL-MC			DNA-MC		
		V3–V4	16S	16S-ITS-23S	V3–V4	16S	16S-ITS-23S
Absolute richness	20	53	29	26	59	22	29
Shannon diversity index	3.00	2.71	2.69	2.54	2.85	2.86	2.71
Pielou’s evenness index	1.00	0.68	0.80	0.78	0.70	0.92	0.80

The RD between determined and theoretical RA provides some insight into the degree of misrepresentation of diversity in V3–V4 sequenced mock communities (Table 5). Consideration of RD values of ≤ − 1.0 or ≥1.0 highlights target species for which there was considerable under and over-representation, respectively. All *Leuconostoc* species were considerably underrepresented in both mock communities (not detected [ND]-0.07% RA), pointing to potential PCR bias (unrelated to DNA copy number) given no closely related species were identified at a RA that could otherwise account for their expected abundance (i.e., misassigned reads), and no improvement was observed when DNA copy number was corrected for in the DNA-MC. A follow-up assessment of the V3–V4 rRNA PCR protocol for target *Leuconostoc* species confirmed very poor amplification in practice (as observed via gel electrophoresis of PCR products; data not shown) despite RibDif2 retrieving 245 amplicons from them collectively using the same primers *in silico*. *Fructilactobacillus fructivorans*, *Latilactobacillus sakei* and *L. salivarius* were considerably overrepresented in the CELL-MC (15.54, 11.03, 11.21% RA, respectively) but this was largely corrected for in the DNA-MC where genome size and estimated DNA copies were taken into consideration (4.44, 8.62, 6.83% RA, respectively). Whilst *Weissella confusa* was underrepresented (ND-0.08% RA), reads were likely misassigned to the known V3–V4 overlapping species (Table 3), *Weissella cibaria* (1.40–4.15% RA).

Other noteworthy diversity discrepancies for V3–V4 sequenced mock communities that could be attributed to the misassignment of reads to known, overlapping species (Table 3), are for the *Lacticaseibacillus* and *Lactiplantibacillus* species. *Lactiplantibacillus paraplantarum* and *L. pentosus* were not detected, however, *L. plantarum* was marginally to considerably overrepresented in CELL-MC (9.65% RA) and DNA-MC (12.87%) samples, suggesting read misassignment may have occurred favoring *L. plantarum*. Similarly, *L. casei* was marginally underrepresented (0.73–1.35% RA) with reads likely misassigned to *L. paracasei* which was considerably overrepresented (16.72–18.89% RA); *Lacticaseibacillus rhamnosus* was not identified as an overlapping species by RibDif2, however, its false identification in CELL-MC (0.93% RA) and DNA-MC (1.15% RA) samples could nevertheless be associated with target *Lacticaseibacillus* species. Although only marginally underrepresented in both mock communities (1.35–1.02% RA), reads for *Liquorilactobacillus mali* were likely misassigned to overlapping species, *Liquorilactobacillus hordei*, which was identified as a false-positive species (2.2–4.3% RA).

Increasing amplicon length to cover the entire 16S rRNA gene notably improved the observed richness and evenness of mock community samples, giving a closer representation of actual diversity (Tables 5, 6). Most importantly, 100 and 95% of target species were correctly identified, and six (1.7% RA) and three (1.0% RA) species were falsely identified in the CELL-MC and DNA-MC, respectively. As a result, community richness (*S* = 29 and 22, respectively) more accurately reflected the expected value of 20. Community evenness also improved, where underrepresented target species decreased to 45% of the CELL-MC and 25% of the DNA-MC compared with the V3–V4 amplified samples. Whilst Shannon diversity indices (*H* = 2.69 and 2.86, respectively) were comparable to that determined for the V3–V4 amplified samples, Pielou’s evenness indices increased favorably for the CELL-MC (*J* = 0.80) and DNA-MC (*J* = 0.92). The high evenness index for the DNA-MC reflects both a lower number of falsely identified, low abundant species and an overall improvement in RA of target species, although *L. pentosus* was not identified.

RibDif2 analysis points to at least one overlap in 16S rRNA amplicons for *Lacticaseibacillus casei*/*paracasei* and *Lactiplantibacillus pentosus*/*plantarum* (Table 3). Mock community 16S rRNA amplicon sequencing showed that *L. plantarum* was well represented in both the CELL-MC (5.81% RA) and DNA-MC (7.00% RA), but *L. pentosus* was considerably underrepresented (0.04% RA) or not detected, respectively. Based on RibDif2 outcomes, it is likely that reads for *L. pentosus* were misassigned to *L. plantarum*, particularly given follow-up assessments of the 16S rRNA PCR protocol confirmed the amplification of the *L. pentosus* isolate used in the mock community (data not shown). *Lacticaseibacillus casei* and *L. paracasei* were both overrepresented in mock community samples despite the identification of allele overlap by RibDif2 (Table 3).

Other instances of misrepresentation of mock community species by 16S rRNA amplicon sequencing revealed by noteworthy RD values included *F. fructivorans* and *L. sakei* which were again considerably overrepresented in the CELL-MC (18.83 and 12.62% RA, respectively) with improvement shown in the DNA-MC with DNA copy correction (6.27 and 9.18% RA, respectively). The RA of *Leuconostoc* species increased for both mock community samples compared to results observed using V3–V4 rRNA amplicons, though were still notably underrepresented in most cases where 16S rRNA amplicons were used (1.37–3.04% RA). Previous observations of probable read misassignment to closely related species of *L. mali* and *W. confusa* with V3–V4 rRNA amplicons were resolved using 16S rRNA amplicons which was consistent with RibDif2 predictions.

Compared with 16S rRNA gene amplicon sequencing, further increase in amplicon length to cover the 16S-ITS-23S rRNA region produced similar descriptions in richness for CELL-MC (*S* = 26) and DNA-MC (*S* = 29) samples, however, evenness (*J* = 0.78 and 0.80, respectively) and diversity (*H* = 2.54 and 2.71, respectively) were lower because of comparably more instances of underrepresentation of target species. All target species were identified, including *L. pentosus*, albeit at considerably low RA (0.05 and 0.12% RA). Follow-up assessments of the 16S-ITS-23S rRNA PCR protocol confirmed *L. pentosus* could be amplified (data not shown), again suggesting reads for *L. pentosus* were misassigned to closely related *L. plantarum*. However, unlike for V3–V4 and 16S rRNA amplicon sequencing, no overlapping alleles were identified between these species by RibDif2 (Table 3). To test the hypothesis that *L. pentosus* reads were nevertheless misassigned to *L. plantarum*, reads were re-analyzed using an amended version of the MIrROR database without *L. plantarum* included (i.e., removal of 376 sequences derived from 125 genomes). Approximately 40% of reads were subsequently re-allocated to *L. pentosus* while the balance were re-allocated to *L. paraplantarum* (data not shown), suggesting a high level of similarity between all target *Lactiplantibacillus* species. This was confirmed using MatGAT software (Campanella et al., 2003) which showed that the average nucleotide identity (ANI) between *L. pentosus* and both *L. plantarum* and *L. paraplantarum* was 97.0 ± 2.8% and 97.0 ± 2.7%, respectively, based on 16S-ITS-23S rRNA amplicons extracted from the MIrROR database. With consideration of the ANI and the average basecall accuracy (96.4%) calculated for reads used in these analyses, it becomes apparent that these values overlap creating an opportunity for reads of these *Lactiplantibacillus* species to be misassigned to one another. In this case, *L. pentosus* reads were almost entirely misassigned, the re-allocation of which appeared to align most often with *L. paraplantarum*, followed by *L. plantarum*.

The most notable discrepancy in the taxonomic assignment of reads in the 16S-ITS-23S rRNA amplified samples was the false identification of *L. zeae.* Given *L. casei* was considerably underrepresented (0.25 and 0.29% RA) in both mock communities, it was hypothesized that *L. casei* reads were misassigned to *L. zeae*. Indeed, by re-analyzing reads without *L. zeae* included in the MIrROR database (i.e., removal of five sequences derived from one genome), ~97% of the reads previously assigned as *L. zeae* were reassigned to *L. casei* (data not shown). Whilst allele overlap was not predicted by RibDif2 analyses with respect to this amplicon, these two species do overlap based on the ITS region in isolation (Table 3). It was further determined using MatGAT software that the ANI between these two species, based on 16S-ITS-23S rRNA sequences in the MIrROR database, was 97.2 ± 2.5%, overlapping with the average basecall accuracy (96.4%) and creating the possibility for *L. casei* reads to be misassigned to *L. zeae*.

Finally, this study included DNA copy-corrected samples to minimize PCR bias and to better attribute observed differences in RA and alpha diversity to the amplicon used. In samples without DNA copy correction, species with smaller genomes would be expected to have more copies of their genome per ng of DNA, resulting in more amplicons during PCR and subsequent presentation of greater abundance in mixed communities. In practice, and largely applying theoretical genome sizes to guide DNA-MC preparation (Table 2), the RA of *F. fructivorans, L. sakei* and *L. salivarius* (V3–V4 and 16S rRNA samples only) decreased in DNA-MC samples suggesting PCR bias connected to DNA copy number played a role in the overrepresentation of these species in CELL-MC samples. This effect was anticipated most obviously for *F. fructivorans* given it has the smallest genome of all target species (1.4 Mb), however, for other species with similarly small genomes (e.g., *L. lactis* and *L. citreum*; ~1.9 Mb), this was not observed. The effect of one source of PCR bias, as previously identified, can offset attempts to correct for others. With an understanding of the role genome size and DNA copy number can play in influencing RA, caution must be exercised in drawing conclusions regarding the true diversity of species in real samples where DNA copy has not been accounted for. Whilst not considered here, multiple copies of the target gene may also affect RA, where species with more copies may be overrepresented in comparison to species with less. It has been previously found, however, that correcting for 16S rRNA gene copy number does not necessarily improve the expected RA of species in mixed communities (Starke et al., 2021). Nevertheless, the 16S rRNA copy number in genomes of the species in our mock community were similar (Table 2) and are therefore not expected to have a notable impact on observed outcomes.

## 4 Discussion

Sequencing of partial, hypervariable regions of the 16S rRNA gene remains the most popular approach for undertaking amplicon-based metataxonomic studies to describe bacterial communities in complex samples (Starke et al., 2021). However, the limitations of this approach are well understood; chiefly, an inability to accurately describe the richness and diversity of these communities at the species-level (Deka et al., 2021; Piraine et al., 2021; Szoboszlay et al., 2023), which has been specifically identified as a limitation for adequately interrogating LAB rich environments to identify species of predicted functional importance and, potentially, economic value (Milani et al., 2018; Deka et al., 2021). Certainly, RibDif2 analysis here demonstrated a greater number of instances where *Lactobacillaceae* species (up to 43) were not distinguished due to at least one V3–V4 rRNA allele overlap compared to when the complete 16S rRNA gene (up to 11), or partial (up to 15)/complete (none) 16S-ITS-23S rRNA regions, were utilized (Table 3). With subsequent amplicon-based metataxonomic profiling of a *Lactobacillaceae* mock community comprising 20 diverse and (some) closely related species, the consequence of allele overlap was demonstrated, and in particular, for V3–V4 rRNA sequenced communities. Compared with sequencing longer rRNA amplicons, V3–V4 rRNA sequenced mock community samples had more: undetected target species (chiefly, *L. paraplantarum* and *L. pentosus*); apparent misassignment of target species’ reads to closely related species (e.g., *L. casei* → *L. paracasei*, *L. paraplantarum/pentosus* → *L. plantarum, L. mali* → *L. hordei, W. confusa* → *W. cibaria*); and under- and overrepresented target species (60–85%).

Similar observations have been made in other studies comparing community profiles derived from V3–V4 rRNA amplicons (and other 16S hypervariable regions) to those based on whole 16S rRNA gene amplicons. Szoboszlay et al. (2023) showed extreme disparity in richness for a 14-species bacterial mock community based on the V4 rRNA region (*S = ~*2,297; Illumina NovaSeq) compared to 16S rRNA (*S = ~*37; ONT), even after various attempts to denoise the V4 data (*S* = ~198). Further, 68% versus 96% of target species were identified, respectively (Szoboszlay et al., 2023), with the authors concluding that 16S rRNA gene amplicon sequencing by Nanopore is the better choice for achieving species-level taxonomic resolution. The same conclusion has been made in other studies investigating complex microbiome samples (Shin et al., 2016; Nygaard et al., 2020; Matsuo et al., 2021; Klair et al., 2023) where greater species-level taxonomic resolution was achieved using 16S rRNA gene amplicon sequencing (ONT) compared to using V3–V4 rRNA (Illumina) amplicons. The outcomes of these studies and the results presented here give cause to exercise caution when drawing conclusions regarding the reported species-level diversity of complex samples that are underpinned by partial 16S rRNA sequencing, especially for studies investigating LAB communities comprised of species with known allele overlap (as presented here) and those identified at low relative abundance.

Amplicon sequencing of the whole 16S rRNA gene best described the diversity of the *Lactobacillaceae* mock community examined in this study, specifically with consideration of the DNA copy-corrected DNA-MC which resulted in the least number of falsely identified species (*n* = 3), the most accurate absolute richness (*S* = 22), and highest evenness (*J* = 0.92) and diversity (*H* = 2.86) indices. The 16S-ITS-23S rRNA amplicon also facilitated an adequate description of DNA-MC diversity (*J* = 0.80 and *H* = 2.71), despite the conspicuous misassignment of *L. casei* reads to *L. zeae*. Few studies have compared the performance of 16S rRNA gene and (complete) 16S-ITS-23S rRNA amplicon sequencing by Nanopore to describe bacterial communities at the species level (Benítez-Páez and Sanz, 2017; Cusco et al., 2019; Kinoshita et al., 2021). A study by Cusco et al. (2019) resulted in similar conclusions to that observed here, where all eight target species were identified in a ZymoBIOMICS mock community using both amplicons, with overall better representation of species abundance using 16S rRNA gene amplicons. In contrast, Kinoshita et al. (2021) examined a 15-species mock community of divergent taxa and showed a reduction in dissimilarity index and average misidentified species (compared to the known community composition) when using 16S-ITS-23S rRNA amplicons (0.23 and 5.3, respectively) compared with 16S rRNA gene amplicons (0.56 and 14.8, respectively). Rozas et al. (2022) also showed an improved species-level description of a 6-species mock community of divergent taxa with 16S-ITS-23S rRNA, albeit using ~2,500 bp amplicons encompassing 16S, ITS and part of the 23S rRNA region.

This study corroborates the conclusion of other studies that Nanopore sequencing of increasingly longer rRNA regions facilitates improved species-level taxonomic classification compared with partial 16S rRNA regions, which can only reliably inform of taxonomic assignment at the genus level or higher. With consideration of workflow efficiencies and availability of comprehensive and well-resourced databases, 16S rRNA gene amplicon sequencing is arguably the current and most prudent choice for amplicon-based bacterial metataxonomics over 16S-ITS-23S rRNA amplicon sequencing, without sacrificing species-level identification confidence. However, as databases improve and as strain-level differentiation becomes necessary, 16S-ITS-23S rRNA sequencing may well find its niche (Benítez-Páez and Sanz, 2017; Seol et al., 2022), especially with continued improvement in Nanopore basecall accuracy.

Basecall accuracy for reads, deduced from Phred scores, can limit our ability to leverage the added discriminatory power of longer rRNA amplicons (Szoboszlay et al., 2023). Indeed, we observed the unanticipated misassignment of 16S-ITS-23S rRNA reads of *L. casei* to *L. zeae* and *L. pentosus* to *L. paraplantarum* and *L. plantarum* and found that the ANI between these species (~97%) overlapped with the average basecall accuracy (96.4%) of our MinION™ sequencing protocol, meaning target species’ reads could be variably assigned to closely related taxon depending on the alignment of reads to database amplicons. It should be pointed out, however, that the identification of allele overlap between species via *in silico* analyses, like RibDif2, does not necessarily mean that these species cannot be distinguished in all circumstances given that each species generally has more than one unique allele; it would only be in cases where the shared allele was the only one amplified from each species during PCR that confusion might arise. The definitive identification of *L. casei* and *L. paracasei* and *Leuconostoc lactis* and *Leuconostoc citreum* in 16S rRNA gene sequenced samples, despite the identification of 16S rRNA gene allele overlap in both cases, supports this. Species diversity and adequate representation of all possible alleles in reference databases is therefore paramount to ensure reads can be correctly assigned (Kinoshita et al., 2021). Further, the use of primers that are genuinely universal and which can amplify all alleles is of equal importance; additional work is required to determine if the ‘universal’ 16S and 16S-ITS-23S rRNA primers used in this study are optimal for all *Lactobacillaceae* and their various alleles.

The *Lactobacillaceae* are a family of LAB that are predominant in a number of environmentally, clinically and industrially important environments, and any improvements in metataxonomic methods that would enable unambiguous, species-level analysis of these communities would be enthusiastically adopted. *In-silico* analysis to identify amplicon overlap (RibDif2) and mock community amplicon sequencing (ONT MinION™) both demonstrate an increasing ability to distinguish diverse and closely related *Lactobacillaceae* species as the rRNA amplicon expands from the less informative V3–V4 hypervariable region to the more informative 16S and 16S-ITS-23S rRNA regions. However, whilst both 16S rRNA gene and 16S-ITS-23S rRNA amplicons were shown to similarly improve the description of diversity of *Lactobacillaceae* communities in practice, there is room for improvement, especially pertaining to the investigation of microbiomes of natural diversity where species richness, abundance, and genome attributes (i.e., size, GC content, gene copy number) will vary considerably, necessitating careful consideration of amplicon library preparation protocols that can manage these. Further, where closely related *Lactobacillaceae* species are expected to be present in mixed communities, irrespective of target amplicon, consideration must still be given to their ANI in the context of basecall accuracy. Oxford Nanopore Technologies currently reports a basecall accuracy of at least 99% when using their R10.4.1 flowcell and V14 kit chemistry (Technologies, Oxford Nanopore, 2022), which should theoretically permit the differentiation of species with an amplicon ANI up to 99%. With such improvements, researchers may benefit more completely from the increased species-level differentiating power held by the whole 16S-ITS-23S rRNA region.

## Data availability statement

The data presented in this study are deposited in an online repository. The repository can be found at https://www.ncbi.nlm.nih.gov/bioproject/1021338, with BioProject accession number PRJNA1021338.

## Author contributions

SO: Conceptualization, Data curation, Formal analysis, Investigation, Methodology, Writing – original draft, Writing – review & editing. MB: Formal analysis, Methodology, Supervision, Writing – review & editing. MS: Software, Writing – review & editing. RM: Software, Writing – review & editing. TR: Formal analysis, Methodology, Supervision, Writing – review & editing. JB: Formal analysis, Methodology, Supervision, Writing – review & editing. BC: Formal analysis, Methodology, Supervision, Writing – review & editing.
